# Immune-Inflammatory, Autophagy, and JNK Remodeling in Ischemic and Nonischemic Human Myocardium

**DOI:** 10.3390/cimb48101082

**Published:** 2026-10-20

**Authors:** Hui-Qi Qu, Joseph Glessner, Dennis McNamara, Michael R. Bristow, Arthur M. Feldman, Hakon Hakonarson

**Affiliations:** 1 Center for Applied Genomics, Children’s Hospital of Philadelphia, Philadelphia, PA 19104, USA; quh@chop.edu (H.-Q.Q.); glessner@chop.edu (J.G.); 2 Department of Pediatrics, Perelman School of Medicine, University of Pennsylvania, Philadelphia, PA 19104, USA; 3 Division of Human Genetics, Children’s Hospital of Philadelphia, Philadelphia, PA 19104, USA; 4 Heart and Vascular Institute, University of Pittsburgh Medical Center, 200 Lothrop Street, Pittsburgh, PA 15213, USA; mcnamaradm@upmc.edu; 5 Department of Medicine, University of Colorado School of Medicine, Anschutz Campus, Aurora, CO 80045, USA; michael.bristow@cuanschutz.edu; 6 Department of Medicine, Lewis Katz School of Medicine, Temple University, Philadelphia, PA 19140, USA; 7 Departments of Neurology and Medicine, Johns Hopkins University School of Medicine, Baltimore, MD 21287, USA; 8 Division of Pulmonary and Sleep Medicine, Children’s Hospital of Philadelphia, Philadelphia, PA 19104, USA; 9 Faculty of Medicine, University of Iceland, 101 Reykjavik, Iceland

**Keywords:** biological state, human heart tissue, RNA sequencing, ischemic cardiomyopathy, nonischemic cardiomyopathy, sex differences, race

## Abstract

This study investigated ischemic versus nonischemic human myocardium across disease phenotypes, sex, and race, prioritizing genes involved in immune-inflammatory signaling, autophagy, JNK-associated stress responses, innate immune recognition, and solute transport. Bulk RNA sequencing was performed in 75 human heart tissue samples, including 13 controls, 18 ischemic samples, and 44 nonischemic samples. Primary models tested ischemic versus control, nonischemic versus control, and nonischemic versus ischemic contrasts. Secondary models evaluated sex- and race-associated expression while adjusting for disease status and sequencing batch. Ischemic versus control comparison identified 3974 genes and nonischemic versus control identified 4276 genes at adjusted *p* < 0.05, while nonischemic versus ischemic identified only two genes. After adjustment for disease status and sequencing batch, 108 genes (including 64 autosomal genes) were associated with sex and 128 with race at FDR < 0.05. In the adjusted race analysis, *SLC39A4* was higher and *MAP1LC3B2* lower in White samples after FDR correction. Ischemic and nonischemic myocardium showed extensive shared remodeling with exploratory nominal differences. Sex and race were associated with additional variation in immune, proteostatic, and nutrient-transport programs. *BAG3* transcript abundance was not significantly altered, consistent with prior evidence that transcript and protein abundance may be uncoupled in failing myocardium.

## 1. Introduction

Ischemic and nonischemic heart disease arise from different initiating injuries but can progress toward shared end-stage myocardial remodeling [1,2]. Sex [3] and race may further shape expression patterns of candidate therapeutic pathways in human heart tissue.

Cardiac disease progression involves immune signaling, proteostasis, autophagy, stress responses, and metabolic regulation. Immune activation can drive myocardial injury and adverse remodeling through cytokine and innate immune signaling [4]. Proteostasis and autophagy regulate damaged proteins and organelles under cardiomyocyte stress [5]. c-Jun N-terminal kinase (JNK) signaling connects cytokine and stress inputs to activator protein 1 (AP-1)-associated transcriptional responses [6]. C-type lectin domain family (CLEC) genes mark innate immune recognition programs [7]. *BAG3* is a cardiac co-chaperone that participates in heat shock protein 70 (HSP70)-dependent protein quality control, autophagy, and stress responses [8]. Experimental loss of *BAG3* disrupts cardiac proteostasis [9]. Our previous study in mice showed that BAG3 protein abundance is reduced by approximately 50% in failing left-ventricular myocardium relative to nonfailing controls [10]. In contrast, human myocardial studies have shown that BAG3 protein abundance varies with heart-failure and genetic context, including reduced protein levels in failing myocardium despite preserved *BAG3* mRNA and variant-specific increases or decreases in BAG3 protein [11]. Solute carrier (SLC) transporter genes may mark altered substrate handling and metabolic state [12]. Heart failure differs by sex in clinical presentation, pathophysiology, and treatment response [13] and by race in disease burden, outcomes, and management [14].

This study investigated transcriptional differences across control, ischemic, and nonischemic human heart tissue, with secondary analyses examining sex- and race-associated differences in cardiac gene-expression patterns. The primary objectives were to identify disease-associated differentially expressed genes (DEGs) and characterize sex- and race-associated differences, particularly within predefined candidate gene categories. For therapeutic targets under active development, including *BAG3*, evaluation in well-characterized human tissue across major biological states can clarify discrepancies between transcript- and protein-level findings.

## 2. Materials and Methods

### 2.1. Human Heart Tissue Cohort

Human heart tissue samples were obtained from a deidentified cardiac tissue collection established through the long-standing collaboration between Dr. Feldman and Dr. Michael R. Bristow (deceased) at the University of Colorado School of Medicine and Dr. Dennis McNamara at the University of Pittsburgh School of Medicine. The collection included failing human left-ventricular myocardium obtained at the time of cardiac transplantation or mechanical circulatory support, as well as nonfailing donor hearts that were unsuitable for transplantation.

The final analysis included 75 samples (20 female and 55 male) after exclusion of one likely duplicate sample. Disease status was available for all samples: 13 control, 18 ischemic, and 44 nonischemic samples. Samples were processed in two batches, and batch was included in the differential-expression models. Race was available for 68 samples based on recorded demographic information (Table 1).

Because age and race were incompletely recorded, these variables were not included as covariates in the primary disease model. Sex distribution was imbalanced across disease groups, especially in the ischemic group. Sex- and race-associated expression differences were evaluated as secondary analyses rather than included as covariates in the primary disease model.

### 2.2. RNA Sequencing and Gene Quantification

Total RNA was extracted from 50 mg of human heart tissue using the RNeasy Mini Kit (QIAGEN, Hilden, Germany) with on-column DNase digestion according to the manufacturer’s protocol. RNA quantity and quality were assessed by spectrophotometry and TapeStation analysis (Agilent Technologies, Santa Clara, CA, USA). RNA-seq libraries were prepared from poly(A)-selected RNA using the TruSeq Stranded mRNA kit (Illumina, San Diego, CA, USA) according to the standard workflow of the Center for Applied Genomics. Quality-controlled libraries were sequenced on an Illumina NovaSeq 6000 with paired-end 101-cycle reads (Illumina, San Diego, CA, USA). The median sequencing depth was 53.88 million input reads per sample, with a range of 30.18–73.83 million.

Sequencing reads were aligned to the human GRCh38 reference genome using STAR v2.5.2b. BAM files were processed using SAMtools v1.16.1, and alignment quality was evaluated using SAMtools flagstat and Qualimap v2.3 [15]. Samples with an overall mapping rate below 80% or a mean mapping quality below 30 were excluded from downstream analysis.

Gene-level read counts were generated using featureCounts from Subread v2.0.2 with the Homo_sapiens.GRCh38.112.gtf annotation. Reads overlapping annotated exons were assigned to genes using the gene_id attribute. Counting was performed in unstranded mode.

### 2.3. Differential Expression Analysis

Gene-level raw counts were analyzed in R v4.3.2 using DESeq2 v1.42.1 [16]. DESeq2 size-factor normalization, dispersion estimation, negative-binomial generalized linear modeling, and Wald tests were applied to the raw count matrix. Benjamini–Hochberg correction was used to control the false discovery rate (FDR) [17]. Genes with very low expression were removed by retaining genes with a total count greater than 1 across samples. For each model, samples missing the variable of interest were excluded from that analysis. Batch was included as an adjustment covariate in all models.

Pairwise contrasts included ischemic versus control, nonischemic versus control, and nonischemic versus ischemic. Sex-associated expression was evaluated using models including sequencing batch, disease status, and sex. Race-associated expression was evaluated using models including sequencing batch, disease status, and race. Samples with missing race information were excluded from race-specific analyses but retained in analyses for which race was not required. Sex × disease and race × disease interaction terms were tested in separate models. Because only one ischemic sample was female, an additional sex-associated analysis was performed within the nonischemic group. The race model compared White versus Black samples. Age was not included in the primary disease model because age information was unavailable for five samples. An age-adjusted sensitivity analysis was performed among the 70 samples with recorded age using models containing sequencing batch, disease status, and age. Normalized counts and variance-stabilizing transformation (VST) expression values were generated using DESeq2. Normalized counts were used to summarize expression magnitude. VST values were used for quality-control visualization, sample-level clustering, and principal component analysis. Principal component analysis (PCA) was performed on VST values to assess sample-level structure by disease status, sequencing batch, sex, and race. For visualization of residual batch structure, batch effects were removed from VST values while preserving disease status.

For the direct nonischemic-versus-ischemic comparison, an approximate power analysis was performed using the observed group sizes and gene-wise expression means and negative-binomial dispersions estimated from the RNA-seq data. The minimum detectable absolute log2 fold change at 80% power and a two-sided α of 0.05 was calculated for each expressed gene and summarized across genes. As a sensitivity analysis, low-expression genes were filtered using edgeR filterByExpr with the default parameters (min.count = 10, min.total.count = 15, large.n = 10, and min.prop = 0.7) and a design matrix including sequencing batch and disease group. The filtered count matrix was then analyzed using the same batch-adjusted DESeq2 model. For genes discussed on the basis of nominal associations, 95% confidence intervals for log2 fold changes were calculated from the model estimates and standard errors.

### 2.4. Candidate Gene Categories

Candidate genes were examined within predefined categories relevant to heart disease and cellular stress response: inflammation/cytokine signaling, autophagy-related genes, c-Jun N-terminal kinase (JNK)-associated genes, C-type lectin domain family (CLEC)/*BAG3*-related genes (selected to assess innate immune-recognition and proteostasis-related stress responses in diseased myocardium), and solute carrier (SLC)/transporter-related genes. Candidate gene results were summarized by comparison and direction of change. The transporter-related category was interpreted broadly because it included multiple SLC genes and was not limited to SLC5A1 and SLC5A2. These candidate categories were defined a priori, independently of the differential-expression results in the present dataset, based on established mechanisms of myocardial remodeling and the investigators’ prior work in heart failure. The categories included 199 inflammation/cytokine genes, 52 autophagy-related genes, 36 JNK-associated genes, 11 CLEC/BAG3-related genes, and 11 SLC/transporter genes. The complete details of each category and the rationale for inclusion are provided in Table S3.

The analysis code and principal pipeline parameters used for quality control, quantification, and differential-expression analysis are available at https://github.com/huiqi-qu/RNAseq-analysis-pipeline (accessed on 17 October 2026).

## 3. Results

### 3.1. Differential Expression by Disease Status, Sex, and Race

**Figure 1:**
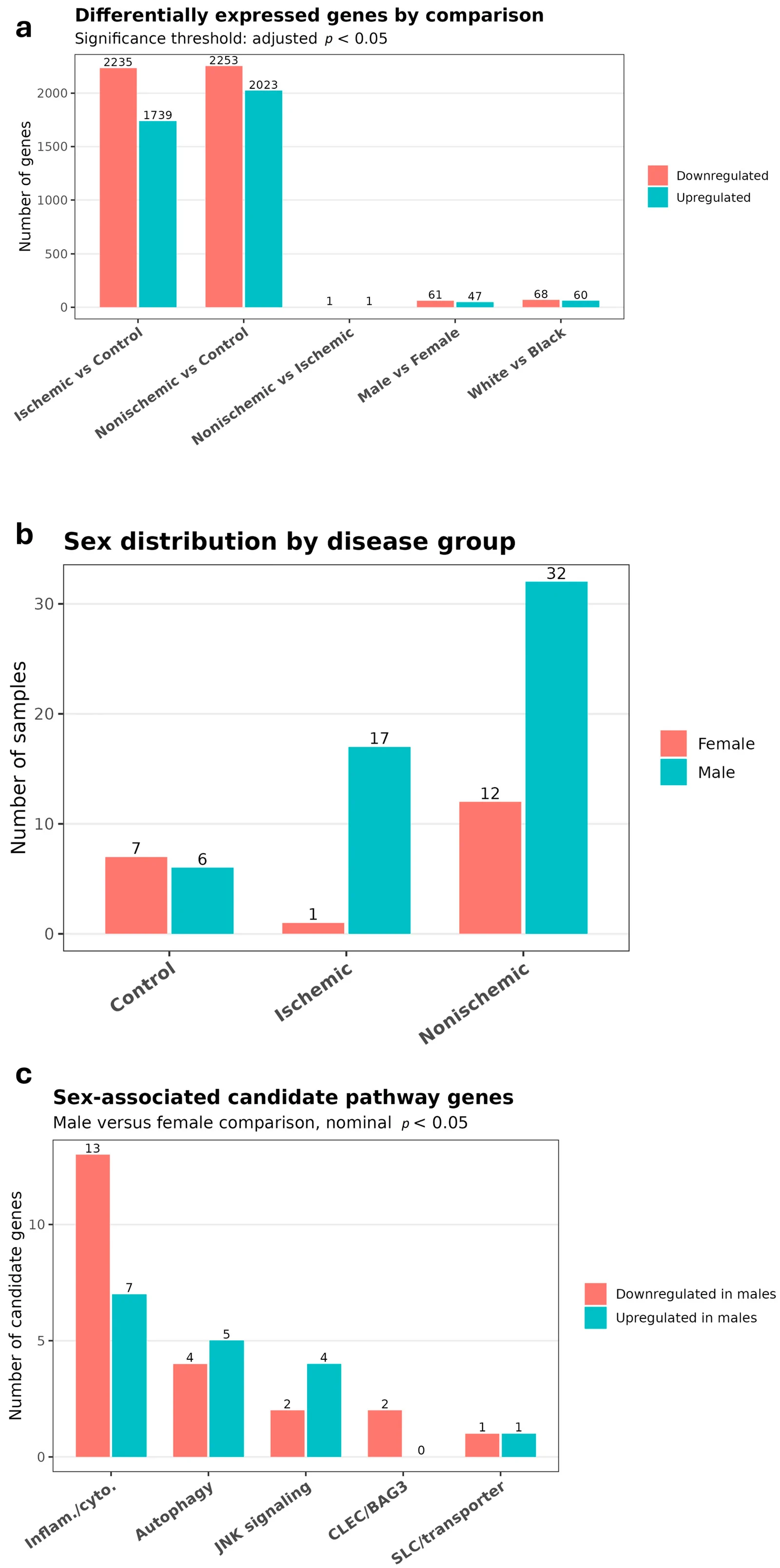
Gene expression comparisons and sex-associated candidate gene analysis. (**a**) Number of upregulated and downregulated genes at adjusted *p* < 0.05 for disease, sex, and race comparisons. (**b**) Female and male sample counts in control, ischemic, and nonischemic groups. (**c**) Candidate genes associated with sex at nominal *p* < 0.05.

Before batch-effect removal, PCA showed pronounced separation by sequencing batch along PC1. This separation was substantially reduced after batch-effect removal (Figure S1). No comparably strong clustering was observed by disease status, sex, or race. A total of 47,871 genes were tested in each DESeq2 comparison (Table 2 and Table S1). The ischemic versus control comparison identified 3974 differentially expressed genes at adjusted *p* < 0.05, including 1739 upregulated and 2235 downregulated genes in ischemic samples relative to controls (Figure 1a). Sex distribution across the three disease groups is shown in Figure 1b. The nonischemic versus control comparison identified 4276 differentially expressed genes, including 2023 upregulated and 2253 downregulated genes in nonischemic samples relative to controls. A total of 3201 genes were significant in both disease–control comparisons, with concordant direction of effect in all cases: 1427 were upregulated and 1774 were downregulated in both ischemic and nonischemic samples relative to controls. In the initial analysis, the nonischemic-versus-ischemic comparison identified two genes at FDR < 0.05, *FAM240A* and *RP11-89N17.3*, both with very low mean expression. Using the more stringent filterByExpr expression filter, 22,868 genes were retained for testing, and no genes remained significant at FDR < 0.05 in the direct nonischemic-versus-ischemic comparison. Log2-fold-change shrinkage also markedly attenuated the extreme effect estimates for the low-expression genes, reducing absolute raw log2 fold changes of approximately 15.8–17.5 to values near zero. At 80% power and a two-sided α of 0.05, the median minimum detectable effect across expressed genes was an absolute log2 fold change of 0.465, corresponding to approximately a 1.38-fold difference. The interquartile range was 0.256–0.938 in absolute log2 fold change, corresponding to approximately 1.19- to 1.92-fold differences.

After adjustment for disease status and sequencing batch, the sex-associated analysis identified 108 genes (64 autosomal) at FDR < 0.05, including 47 with higher and 61 with lower expression in males (Figure 1a). In the nonischemic-only sensitivity analysis, 74 genes were associated with sex at FDR < 0.05, and all showed the same direction of effect in the full disease-adjusted sex model. The sex × disease interaction analysis identified 5 genes at FDR < 0.05. The race-associated analysis identified 128 genes at FDR < 0.05, including 60 with higher and 68 with lower expression in White relative to Black samples (Figure 1a). The race × disease interaction analysis identified 41 genes at FDR < 0.05.

In the Batch-1-only sensitivity analysis, genome-wide log2 fold-change estimates were correlated with those from the full batch-adjusted model, with Spearman correlations of 0.718 for ischemic versus control, 0.760 for nonischemic versus control, and 0.789 for nonischemic versus ischemic. Directional concordance among genes significant in the full-model analyses was 98.2% for ischemic versus control and 99.8% for nonischemic versus control. Effect estimates for representative genes before and after adjustment for sequencing batch are shown in Table S2. In an age-adjusted sensitivity analysis restricted to the 70 samples with recorded age, disease-associated effect estimates were highly concordant with those from corresponding models without age adjustment. Spearman correlations of log2 fold-change estimates were 0.985 for ischemic versus control, 0.989 for nonischemic versus control, and 0.972 for nonischemic versus ischemic, with directional concordance across all genes of 97.2%, 98.4%, and 96.1%, respectively.

### 3.2. Candidate-Gene Analysis

Candidate-gene analyses used predefined inflammation/cytokine, autophagy, JNK, CLEC/*BAG3*, and SLC/transporter gene sets (Tables S3 and S4).

### 3.3. Inflammation/Cytokine Genes Show Extensive Shared Remodeling

Inflammation/cytokine genes formed the largest candidate category in both disease–control comparisons. In ischemic versus control, 65 genes were nominally significant, including 19 upregulated and 46 downregulated genes. A total of 41 remained significant after FDR correction, including 10 upregulated and 31 downregulated genes. In nonischemic versus control, 60 genes were nominally significant, including 18 upregulated and 42 downregulated genes. Of those, 46 remained significant after FDR correction, including 15 upregulated and 31 downregulated genes. A total of 53 nominally significant genes were shared between the 2 disease–control comparisons. All showed concordant direction of effect, including 17 genes upregulated and 36 genes downregulated in both disease groups. There were 37 FDR-significant genes that were shared, including 10 concordantly upregulated and 27 concordantly downregulated genes. In nonischemic versus ischemic, 12 inflammation/cytokine genes were nominally significant, including 9 upregulated and 3 downregulated genes in nonischemic samples. None remained significant after FDR correction.

### 3.4. Autophagy-Related Genes Show Concordant Disease-Associated Changes

In ischemic versus control, 22 autophagy-related genes were nominally significant, including 8 upregulated and 14 downregulated genes. A total of 16 remained significant after FDR correction, including 6 upregulated and 10 downregulated genes. In nonischemic versus control, 21 autophagy-related genes were nominally significant, including 7 upregulated and 14 downregulated genes. There were 15 that remained significant after FDR correction, including 6 upregulated and 9 downregulated genes. In total, 20 nominally significant autophagy-related genes were shared between the 2 disease–control comparisons. All showed concordant direction of effect, including 7 genes upregulated and 13 genes downregulated in both disease groups. All 15 FDR-significant autophagy-related genes in the nonischemic versus control comparison were also significant in ischemic versus control, with 6 concordantly upregulated and 9 concordantly downregulated genes. In nonischemic versus ischemic, an autophagy-related gene *SLC36A4* was nominally upregulated in nonischemic samples. No autophagy-related genes remained significant after FDR correction.

### 3.5. JNK-Associated Genes Show Shared Bidirectional Remodeling

In ischemic versus control, 20 JNK-associated genes were nominally significant, including 13 upregulated and 7 downregulated genes. 11 remained significant after FDR correction, including 7 upregulated and 4 downregulated genes. In nonischemic versus control, 20 JNK-associated genes were nominally significant, including 13 upregulated and 7 downregulated genes. 13 remained significant after FDR correction, including 9 upregulated and 4 downregulated genes. 17 nominally significant JNK-associated genes were shared between the disease–control comparisons. All showed concordant direction of effect, including 11 genes upregulated and 6 genes downregulated in both disease groups. 9 FDR-significant genes were shared, including 6 concordantly upregulated and 3 concordantly downregulated genes. Selected upregulated JNK-associated genes included *JUN* and *MAP3K7* in ischemic samples and *ATF2*, *MAPK8*, and *MAP3K7* in nonischemic samples. 1 JNK-associated gene was nominally upregulated in nonischemic versus ischemic. No JNK-associated genes remained significant after FDR correction.

### 3.6. CLEC-Family Genes Show a Smaller Shared Disease Signal

In ischemic versus control, 7 CLEC genes were nominally significant, all of which were downregulated. 4 remained significant after FDR correction: *CLEC10A*, *CLEC2B*, *CLEC4D*, and *CLEC7A*. In nonischemic versus control, 6 CLEC genes were nominally significant, including 1 upregulated and 5 downregulated genes. 4 downregulated genes remained significant after FDR correction: *CLEC10A*, *CLEC16A*, *CLEC5A*, and *CLEC7A*. 5 nominally significant CLEC genes were shared between the disease–control comparisons, and all were downregulated in both disease groups. 2 FDR-significant genes were shared, both with concordant downregulation. In nonischemic versus ischemic, *CLEC4D* was nominally upregulated in nonischemic samples. It did not remain significant after FDR correction. *BAG3* was not significantly differentially expressed in either disease–control comparison. In ischemic versus control myocardium, *BAG3* showed a log2 fold change of −0.263 (95% CI −0.688 to 0.162; adjusted *p* = 0.454), with mean normalized expression of 11,639.4 counts in ischemic samples and 13,555.2 counts in controls. In nonischemic versus control myocardium, *BAG3* showed a log2 fold change of −0.034 (95% CI −0.429 to 0.361; adjusted *p* = 0.936), with mean normalized expression of 14,262.8 counts in nonischemic samples and 13,555.2 counts in controls. *CLEC16A* was nominally downregulated in ischemic versus control and FDR significantly downregulated in nonischemic versus control.

### 3.7. SLC/Transporter Genes Show Concordant Downregulation

In ischemic versus control, 6 SLC/transporter genes were nominally significant, all of which were downregulated. Of those, 5 remained significant after FDR correction: *SLC50A1*, *SLC51A*, *SLC52A2*, *SLC52A3*, and *SLC5A6*. In nonischemic versus control, 7 SLC/transporter genes were nominally significant, including 1 upregulated and 6 downregulated genes. In total, 6 downregulated genes remained significant after FDR correction: *SLC50A1*, *SLC51A*, *SLC52A2*, *SLC52A3*, *SLC5A10*, and *SLC5A6*. *SLC5A7* was nominally upregulated but did not remain significant after FDR correction. There were 6 nominally significant SLC/transporter genes that were shared between the disease–control comparisons, and all were downregulated in both disease groups. A total of 5 FDR-significant genes were shared, also with concordant downregulation. No SLC/transporter genes were nominally or FDR significant in nonischemic versus ischemic.

### 3.8. Sex-Associated Genes

In male versus female, 20 inflammation/cytokine genes were nominally significant, including 7 with higher and 13 with lower expression in males (Figure 1c). In total, 9 autophagy-related genes were nominally significant, including 5 with higher and 4 with lower expression in males. A total of 6 JNK-associated genes were nominally significant, including 4 with higher and 2 with lower expression in males. There were 2 CLEC/*BAG3* genes that were nominally significant, both with lower expression in males. A total of 2 SLC/transporter genes were nominally significant, including 1 with higher and 1 with lower expression in males. No candidate genes in these categories remained significant after FDR correction. *CXCL6* was nominally lower in males, and *GABARAPL1* was nominally higher in males. Per-sample normalized counts showed sparse *CXCL6* expression, with a median of 5.51 in females and 0 in males; 5 of 20 female samples and 31 of 55 male samples had zero counts (Figure S2).

### 3.9. Race-Associated Genes

In White versus Black, 26 inflammation/cytokine genes were nominally significant, including 3 upregulated and 23 downregulated genes in White samples. None remained significant after FDR correction. In total, 11 autophagy-related genes were nominally significant, including 4 upregulated and 7 downregulated genes. Of those, 2 remained significant after FDR correction: *SLC39A4* was upregulated and *MAP1LC3B2* was downregulated in White samples. There were 8 JNK-associated genes that were nominally significant, including 4 upregulated and 4 downregulated genes. In total, 2 CLEC genes, *CLEC18C* and *CLEC4F*, were nominally upregulated. There were 3 SLC/transporter genes that were nominally significant, including 2 upregulated and 1 downregulated gene. No inflammation/cytokine, JNK-associated, CLEC/*BAG3*, or SLC/transporter genes remained significant after FDR correction.

## 4. Discussion

### 4.1. Convergent Remodeling in Ischemic and Nonischemic Myocardium

Ischemic and nonischemic myocardium showed extensive shared transcriptional remodeling relative to control tissue. The direct comparison identified nominal etiology-specific expression differences. These candidate-gene associations are considered exploratory and hypothesis-generating. Previous studies have shown substantial convergence of gene-expression changes in advanced heart failure [1,2].

Each disease–control comparison contained candidate genes that were not significant in the other, particularly within the inflammation/cytokine and JNK-associated categories. The direct comparison also identified nominal differences involving cytokine ligands and receptors, including *TNF*, *IL23A*, *TNFSF14*, and *TNFRSF8*. Although these effects did not remain significant after FDR correction, the nominal differences are consistent with partly different inflammatory inputs in ischemic and nonischemic myocardium. Ischemic injury is initiated by impaired perfusion, tissue necrosis, and repeated ischemic stress [18], whereas nonischemic disease encompasses heterogeneous mechanical, genetic, toxic, and inflammatory insults [19].

### 4.2. Immune-Inflammatory Signaling

In ischemic and nonischemic myocardium, interferon-responsive genes, including *CXCL9*, *IFI44*, *IFI44L*, *IFIH1*, and *IFIT3*, were increased relative to controls. Several cytokine receptors and proximal innate immune signaling genes, including *CCR1*, *CCR2*, *IL1R1*, *IL6R*, *IRAK1*, *IRAK4*, *JAK1*, *MYD88*, and *TGFBR2*, were reduced. Persistent myocardial injury may sustain interferon-associated transcription while altering cytokine responsiveness and immune-cell composition. Inflammatory responses in the injured heart are cell- and stage-dependent and contribute to cardiomyocyte injury, clearance of damaged tissue, repair, fibrosis, and ventricular remodeling [4,20].

The nonischemic-versus-ischemic comparison identified several exploratory nominal associations involving inflammatory genes. Compared with ischemic myocardium, nonischemic myocardium showed nominally higher expression of *TNF*, *IL23A*, *TNFSF14*, and *TNFRSF8* and nominally lower expression of *TNFSF18*, *IL11RA*, and *TNFRSF25*. None remained significant after FDR correction, and these findings are considered hypothesis-generating. Previous human myocardial transcriptomic studies have identified both common end-stage remodeling and etiology-associated immune signals in ischemic and nonischemic heart failure [1,2]. In the present dataset, *TNF* was significantly altered in nonischemic versus control myocardium but not in ischemic versus control myocardium, and the direct nonischemic-versus-ischemic difference did not remain significant after FDR correction. *TNF* has been detected in both ischemic and dilated failing human myocardium, and cardiac-specific *TNF* overexpression produces progressive cardiomyopathy and heart failure in experimental models [21,22]. Circulating *TNF* levels have also been associated with heart-failure severity and mortality [23].

### 4.3. Autophagy-Related Remodeling

Autophagy supports cardiomyocyte proteostasis and organelle quality control under energetic and mechanical stress [5]. In ischemic and nonischemic myocardium, *ATG10*, *ATG14*, *ATG2B*, *GABARAPL1*, *PIK3C3*, and *ULK2* were increased relative to controls, while *ATG4D*, *ATG9A*, *EI24*, *MAP1LC3B*, *TFE3*, and several transporter-related genes were reduced. The opposing changes involved genes participating in autophagosome formation, cargo processing, and lysosomal control during myocardial remodeling [24].

The disease groups showed nominally etiology-associated differences. Compared with ischemic myocardium, nonischemic myocardium showed nominally higher *SLC36A4* expression. *SLC36A4* encodes a solute carrier involved in intracellular nutrient and metabolite handling [25,26].

### 4.4. JNK-Associated Signaling

In ischemic myocardium, *JUN* and *MAP3K7* were increased relative to controls. In nonischemic myocardium, *ATF2*, *MAPK8*, and *MAP3K7* were increased. *MAP3K14* and *JUP* were reduced in both disease groups. The affected genes include stress-activated kinases and JNK-responsive transcriptional regulators involved in AP-1-associated transcription [6]. JNK signaling is activated by inflammatory and cellular stress and can produce adaptive or maladaptive cardiac responses according to the cellular context and duration of signaling [27].

Compared with ischemic myocardium, nonischemic myocardium showed nominally higher *MAPK8IP2* expression. *MAPK8IP2* encodes JIP2, a scaffold that organizes JNK-pathway kinases and regulates the localization and transmission of signals through the kinase cascade [28]. This nominal difference may indicate changes in the spatial organization of JNK signaling, although the association did not remain significant after FDR correction and is considered exploratory.

### 4.5. Cellular Stress-Response

CLEC-family genes were predominantly reduced in diseased myocardium. *CLEC10A* and *CLEC7A* were reduced in both disease groups. *CLEC2B* and *CLEC4D* were additionally reduced in ischemic myocardium, while *CLEC16A* and *CLEC5A* were reduced in nonischemic myocardium. CLEC-family receptors regulate innate immune recognition and leukocyte responses. The observed differences may reflect changes in myocardial immune activation [7].

SLC/transporter genes were consistently reduced across disease groups. *SLC50A1*, *SLC51A*, *SLC52A2*, *SLC52A3*, and *SLC5A6* were reduced in ischemic and nonischemic myocardium, while *SLC5A10* was additionally reduced in nonischemic myocardium. These genes mediate the transport of sugars, bile acid-related molecules, riboflavin, and vitamins [12].

*BAG3* transcript abundance was not significantly altered in either disease group. Protein abundance was not measured in the present study due to limited residual tissue. A previous study of failing human myocardium similarly found no significant difference in *BAG3* mRNA despite lower BAG3 protein levels in the overall ischemic and idiopathic dilated cardiomyopathy groups [11]. BAG3 protein levels also varied among hearts carrying different *BAG3* variants, indicating that transcript abundance alone does not define the BAG3 protein phenotype. Altered proteasomal handling may reduce functional BAG3 despite preserved transcript abundance. BAG3 protein stability is also regulated through its interaction with HSP70; pharmacological inhibition of this interaction in cardiomyocytes reduced the measured BAG3 protein half-life from approximately 28 h to 3 h [29].

### 4.6. Sex-Associated Immune and Proteostatic Profiles

Male and female myocardium differed across immune-inflammatory, autophagy, JNK, CLEC, and transporter genes. Inflammation/cytokine genes were predominantly lower in males, with *CXCL6* nominally lower in males. *CXCL6* encodes a neutrophil-recruiting chemokine [30], and its lower expression in males may reflect reduced chemokine production or neutrophil recruitment. Other nominally lower genes in males included chemokine receptors, innate immune regulators, and cytokine-related genes.

Autophagy-related genes changed in both directions. *GABARAPL1* was nominally higher in males and has a role in autophagic vesicle biology [31]. Nominal differences involved *ATG14*, *ULK1*, *GABARAP*, *EI24*, and transporter-associated genes related to autophagosome formation, cargo processing, and substrate handling [32].

JNK-associated, CLEC-family, and transporter genes differed by sex. Previous studies have shown that male and female hearts differ in transcriptional responses to ischemic stress and in pathways contributing to cardiac remodeling and heart-failure progression [3,13]. These findings indicate that sex may influence responses to pathway-directed therapies [33].

### 4.7. Race-Associated Immune and Nutrient-Transport Remodeling

In White myocardium, lower expression of multiple interferon-responsive, cytokine, NF-κB-regulatory, and innate immune genes indicates reduced activity of selected inflammatory signaling relative to Black myocardium. Within the autophagy-related category, *SLC39A4* was higher and *MAP1LC3B2* was lower in White myocardium after FDR correction. *SLC39A4* regulates cellular zinc uptake [34], which contributes to micronutrient availability, redox control, and immune signaling. Lower *MAP1LC3B2* expression may affect LC3-family autophagy machinery [35].

*IFI44L*, a type I interferon-stimulated gene, was nominally lower in White than Black samples. *IFI44L* acts as a negative-feedback regulator of type I interferon signaling, helping limit excessive antiviral and innate immune activation [36].

### 4.8. External Validation and Study Limitations

We evaluated the candidate-gene findings in the previously published GSE57338 cohort, which includes left-ventricular myocardium from 95 patients with ischemic heart failure, 82 patients with idiopathic dilated cardiomyopathy, and 136 non-failing controls [37]. Gene expression in this cohort was measured using the Affymetrix Human Gene 1.1 ST Array. Of the 309 candidate genes examined in the present study, 291 were represented in GSE57338. Among candidate-gene associations that were nominally significant in the present study, 92 ischemic-versus-control and 89 nonischemic-versus-control associations were also nominally significant in GSE57338 and showed the same direction of effect. In contrast, only two nominal nonischemic-versus-ischemic associations met these criteria. Overall, 183 gene-comparison associations involving 107 unique genes showed nominal significance and concordant direction in both datasets (Table S5). These findings provide independent support for a substantial portion of the candidate-gene changes observed in failing versus control myocardium.

Incomplete replication between the two studies is expected given differences in both study populations and experimental platforms. GSE57338 used an Affymetrix microarray, whereas the present study used RNA sequencing, with differences in transcript coverage, dynamic range, probe representation, and quantification. Differences in disease severity, clinical characteristics, treatment exposure, tissue procurement, and cellular composition may also contribute to variation in myocardial gene expression between cohorts. In addition, many of the candidate-gene findings evaluated here were nominal associations with modest effect sizes and would be expected to be less consistently reproduced across independent datasets.

Our study has several limitations. Control myocardium was derived from donor hearts that were unsuitable for transplantation. Donor physiology, including brain death, critical illness, medication exposure, and peri-procurement inflammatory responses, may influence myocardial gene expression. Differences between failing and control myocardium, particularly immune-related expression differences, may reflect both disease biology and tissue-source effects. Bulk RNA sequencing cannot separate gene-expression changes within cardiomyocytes, fibroblasts, endothelial cells, vascular cells, and immune cells from differences in the relative abundance of these cell populations. The observed immune-inflammatory and CLEC-family differences may reflect changes in immune-cell abundance, transcriptional activity, or both. Nominal findings from the direct disease, sex, and race comparisons should be considered hypothesis-generating and require further replication. Sex and race were unevenly distributed across disease groups. Sequencing batch was unevenly distributed across disease groups. Although batch was included as a covariate and the Batch-1-only sensitivity analysis showed substantial directional concordance with the full batch-adjusted analyses, the unbalanced design may limit complete separation of technical and biological effects. Residual confounding remains possible, particularly because information on social determinants of health, healthcare access, socioeconomic conditions, treatment history, and related factors was not available. RNA expression does not necessarily reflect protein abundance or functional activity, including kinase signaling, receptor responses, transporter activity, and autophagic flux.

## 5. Conclusions

Advanced ischemic and nonischemic heart failure converge at the level of myocardial remodeling, while retaining nominal differences in the upstream signals. Coordinated changes were observed across immune signaling, autophagy, JNK-associated stress responses, innate immune recognition, and nutrient transport. These etiology-associated differences should be considered exploratory because they did not remain significant after FDR correction. *BAG3* transcript abundance was not significantly altered in both disease groups, consistent with previous reports of reduced *BAG3* protein despite preserved transcript abundance in failing myocardium. Sex and race were associated with additional differences in immune, proteostatic, and nutrient-transport pathways.

## Figures and Tables

**Table 1:** Cohort and sample characteristics.

Characteristic	Control	Ischemic	Nonischemic
*N*	13	18	44
Age, years, mean ± SD	53.8 ± 5.2	55.3 ± 9.3	51.0 ± 15.2
Age, years, median (range)	55.5 (42–58)	56.0 (34–68)	58.0 (18–70)
Age missing, *n*	5	0	0
Female, *n* (%)	7 (53.8%)	1 (5.6%)	12 (27.3%)
Male, *n* (%)	6 (46.2%)	17 (94.4%)	32 (72.7%)
Black, *n* (%)	4 (30.8%)	5 (27.8%)	13 (29.5%)
White, *n* (%)	4 (30.8%)	13 (72.2%)	29 (65.9%)
Race missing, *n*	5	0	2
Batch 1, *n* (%)	5 (38.5%)	11 (61.1%)	38 (86.4%)
Batch 2, *n* (%)	8 (61.5%)	7 (38.9%)	6 (13.6%)

**Table 2:** Differentially expressed genes.

Comparison	Total Genes Tested	Significant Genes, Adjusted *p* < 0.05	Upregulated	Downregulated	Minimum Adjusted *p*	Strongest Positive log_2_FC	Strongest Negative log_2_FC
Ischemic vs. Control	47,871	3974	1739	2235	2.30 × 10^−17^	5.04	−7.03
Nonischemic vs. Control	47,871	4276	2023	2253	4.78 × 10^−21^	4.56	−5.44
Nonischemic vs. Ischemic	47,871	2	1	1	7.18 × 10^−12^	18.00	−17.30
Male vs. Female	47,871	108	47	61	0	20.85	−30.00
White vs. Black	47,871	128	60	68	4.54 × 10^−22^	3.69	−6.17

## Data Availability

The RNA-sequencing data generated in this study have now been deposited in the Gene Expression Omnibus (GEO; accession no. GSE349247) and will be released upon publication of the paper. Additional supporting data are available from the corresponding author, Hakon Hakonarson, upon reasonable request.

## Supplementary Materials

The following supporting information can be downloaded at https://www.mdpi.com/article/10.3390/cimb48101082/s1.

## Abbreviations

The following abbreviations are used in this manuscript:

AP-1	Activator protein 1
CLEC	C-type lectin domain family
DEG	Differentially expressed gene
FDR	False discovery rate
HSP70	Heat shock protein 70
JNK	c-Jun N-terminal kinase
RNA-seq	RNA sequencing
SLC	Solute carrier
VST	Variance-stabilizing transformation

## References

[1] Sweet M.E., Cocciolo A., Slavov D., Jones K.L., Sweet J.R., Graw S.L., Reece T.B., Ambardekar A.V., Bristow M.R., Mestroni L. (2018). Transcriptome analysis of human heart failure reveals dysregulated cell adhesion in dilated cardiomyopathy and activated immune pathways in ischemic heart failure. BMC Genom..

[2] Simonson B., Chaffin M., Hill M.C., Atwa O., Guedira Y., Bhasin H., Hall A.W., Hayat S., Baumgart S., Bedi K.C. (2023). Single-nucleus RNA sequencing in ischemic cardiomyopathy reveals common transcriptional profile underlying end-stage heart failure. Cell Rep..

[3] Stone G., Choi A., Meritxell O., Gorham J., Heydarpour M., Seidman C.E., Seidman J.G., Aranki S.F., Body S.C., Carey V.J. (2019). Sex differences in gene expression in response to ischemia in the human left ventricular myocardium. Hum. Mol. Genet..

[4] Mann D.L. (2015). Innate immunity and the failing heart: The cytokine hypothesis revisited. Circ. Res..

[5] Mizushima N., Komatsu M. (2011). Autophagy: Renovation of cells and tissues. Cell.

[6] Davis R.J. (2000). Signal transduction by the JNK group of MAP kinases. Cell.

[7] Scur M., Parsons B.D., Dey S., Makrigiannis A.P. (2023). The diverse roles of C-type lectin-like receptors in immunity. Front. Immunol..

[8] Qu H.Q., Feldman A.M., Hakonarson H. (2022). Genetics of BAG3: A paradigm for developing precision therapies for dilated cardiomyopathies. J. Am. Heart Assoc..

[9] Fang X., Bogomolovas J., Wu T., Zhang W., Liu C., Veevers J., Stroud M.J., Zhang Z., Ma X., Mu Y. (2017). Loss-of-function mutations in co-chaperone BAG3 destabilize small HSPs and cause cardiomyopathy. J. Clin. Investig..

[10] Wang J., Tomar D., Martin T.G., Dubey S., Dubey P.K., Song J., Landesberg G., McCormick M.G., Myers V.D., Merali S. (2023). Bag3 Regulates Mitochondrial Function and the Inflammasome Through Canonical and Noncanonical Pathways in the Heart. JACC Basic Transl. Sci..

[11] Myers V.D., Gerhard G.S., McNamara D.M., Tomar D., Madesh M., Kaniper S., Ramsey F.V., Fisher S.G., Ingersoll R.G., Kasch-Semenza L. (2018). Association of Variants in BAG3 with Cardiomyopathy Outcomes in African American Individuals. JAMA Cardiol..

[12] Khan A., Liu Y., Gad M., Kenny T.C., Birsoy K. (2025). Solute carriers: The gatekeepers of metabolism. Cell.

[13] Lam C.S., Arnott C., Beale A.L., Chandramouli C., Hilfiker-Kleiner D., Kaye D.M., Ky B., Santema B.T., Sliwa K., Voors A.A. (2019). Sex differences in heart failure. Eur. Heart J..

[14] Lewsey S.C., Breathett K. (2021). Racial and ethnic disparities in heart failure: Current state and future directions. Curr. Opin. Cardiol..

[15] Li H., Handsaker B., Wysoker A., Fennell T., Ruan J., Homer N., Marth G., Abecasis G., Durbin R. (2009). 1000 Genome Project Data Processing Subgroup. The sequence alignment/map format and SAMtools. Bioinformatics.

[16] Love M.I., Huber W., Anders S. (2014). Moderated estimation of fold change and dispersion for RNA-seq data with DESeq2. Genome Biol..

[17] Benjamini Y., Hochberg Y. (1995). Controlling the false discovery rate: A practical and powerful approach to multiple testing. J. R. Stat. Soc. Ser. B Methodol..

[18] Heusch G. (2020). Myocardial ischaemia–reperfusion injury and cardioprotection in perspective. Nat. Rev. Cardiol..

[19] Schultheiss H.-P., Fairweather D., Caforio A.L., Escher F., Hershberger R.E., Lipshultz S.E., Liu P.P., Matsumori A., Mazzanti A., McMurray J. (2019). Dilated cardiomyopathy. Nat. Rev. Dis. Primers.

[20] Frangogiannis N.G. (2014). The inflammatory response in myocardial injury, repair, and remodelling. Nat. Rev. Cardiol..

[21] Saba S., Janczewski A.M., Baker L.C., Shusterman V., Gursoy E.C., Feldman A.M., Salama G., McTiernan C.F., London B. (2005). Atrial contractile dysfunction, fibrosis, and arrhythmias in a mouse model of cardiomyopathy secondary to cardiac-specific overexpression of tumor necrosis factor-{alpha}. Am. J. Physiol. Heart Circ. Physiol..

[22] Torre-Amione G., Kapadia S., Lee J., Durand J.B., Bies R.D., Young J.B., Mann D.L. (1996). Tumor necrosis factor-alpha and tumor necrosis factor receptors in the failing human heart. Circulation.

[23] Dunlay S.M., Weston S.A., Redfield M.M., Killian J.M., Roger V.L. (2008). Tumor necrosis factor-alpha and mortality in heart failure: A community study. Circulation.

[24] Dikic I., Elazar Z. (2018). Mechanism and medical implications of mammalian autophagy. Nat. Rev. Mol. Cell Biol..

[25] Pillai S.M., Meredith D. (2011). SLC36A4 (hPAT4) is a high affinity amino acid transporter when expressed in Xenopus laevis oocytes. J. Biol. Chem..

[26] Ma X., Xu S. (2022). Archaic introgression contributed to the pre-agriculture adaptation of vitamin B1 metabolism in East Asia. iScience.

[27] Rose B.A., Force T., Wang Y. (2010). Mitogen-activated protein kinase signaling in the heart: Angels versus demons in a heart-breaking tale. Physiol. Rev..

[28] Whitmarsh A.J. (2006). The JIP family of MAPK scaffold proteins. Biochem. Soc. Trans..

[29] Martin T.G., Delligatti C.E., Muntu N.A., Stachowski-Doll M.J., Kirk J.A. (2022). Pharmacological inhibition of BAG3-HSP70 with the proposed cancer therapeutic JG-98 is toxic for cardiomyocytes. J. Cell. Biochem..

[30] Dai C.-L., Yang H.-X., Liu Q.-P., Rahman K., Zhang H. (2023). CXCL6: A potential therapeutic target for inflammation and cancer. Clin. Exp. Med..

[31] Chakrama F.Z., Seguin-Py S., Le Grand J.N., Fraichard A., Delage-Mourroux R., Despouy G., Perez V., Jouvenot M., Boyer-Guittaut M. (2010). GABARAPL1 (GEC1) associates with autophagic vesicles. Autophagy.

[32] Wollert T. (2019). Autophagy. Curr. Biol..

[33] Mauvais-Jarvis F., Merz N.B., Barnes P.J., Brinton R.D., Carrero J.-J., DeMeo D.L., De Vries G.J., Epperson C.N., Govindan R., Klein S.L. (2020). Sex and gender: Modifiers of health, disease, and medicine. Lancet.

[34] Eide D.J. (2004). The SLC39 family of metal ion transporters. Pflügers Arch..

[35] Schaaf M.B., Keulers T.G., Vooijs M.A., Rouschop K.M. (2016). LC3/GABARAP family proteins: Autophagy-(un) related functions. FASEB J..

[36] DeDiego M.L., Martinez-Sobrido L., Topham D.J. (2019). Novel functions of IFI44L as a feedback regulator of host antiviral responses. J. Virol..

[37] Liu Y., Morley M., Brandimarto J., Hannenhalli S., Hu Y., Ashley E.A., Tang W.H., Moravec C.S., Margulies K.B., Cappola T.P. (2015). RNA-Seq identifies novel myocardial gene expression signatures of heart failure. Genomics.

