# Supplementary material for: Immune-Inflammatory, Autophagy, and JNK Remodeling in Ischemic and Nonischemic Human Myocardium
Source: Curr Issues Mol Biol. 2026 Oct 20;48(10):1082. doi: 10.3390/cimb48101082 (PMC13703238; doi:10.3390/cimb48101082)
Supplement: Supplementary file 1 [file cimb-48-01082-s001.zip › FigS1.pdf]

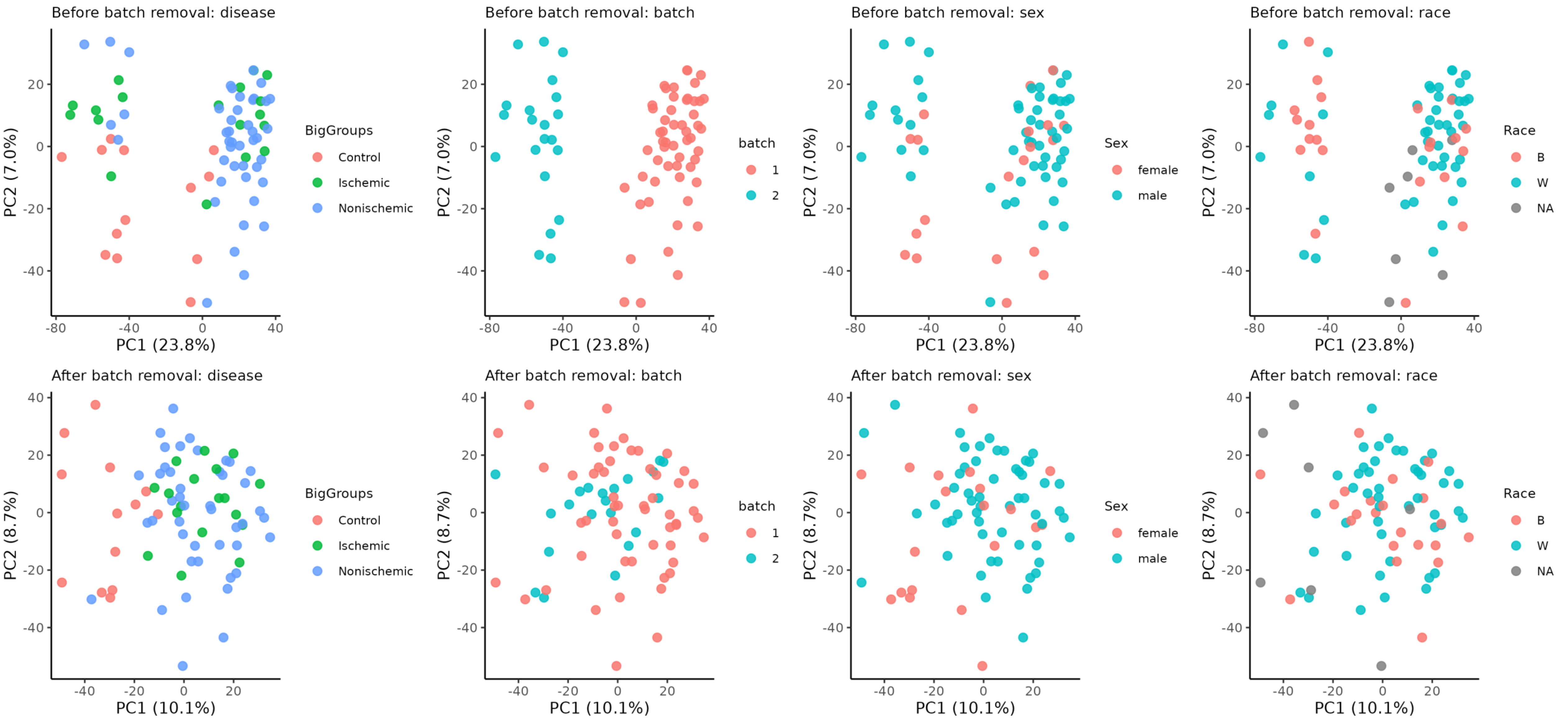

Figure S1. Principal component analysis before and after batch-effect removal. Principal component analysis was performed using variance-stabilized RNA-seq expression values. Samples are shown before batch-effect removal (top row) and after batch-effect removal for visualization using removeBatchEffect while preserving disease-group effects (bottom row). Samples are colored by disease status, sequencing batch, sex, and race, respectively. The percentages shown on the axes indicate the proportion of variance explained by PC1 and PC2.
