# Supplementary material for: Immune-Inflammatory, Autophagy, and JNK Remodeling in Ischemic and Nonischemic Human Myocardium
Source: Curr Issues Mol Biol. 2026 Oct 20;48(10):1082. doi: 10.3390/cimb48101082 (PMC13703238; doi:10.3390/cimb48101082)
Supplement: Supplementary file 1 [file cimb-48-01082-s001.zip › FigS2_CXCL6_per_sample_normalized_counts.pdf]

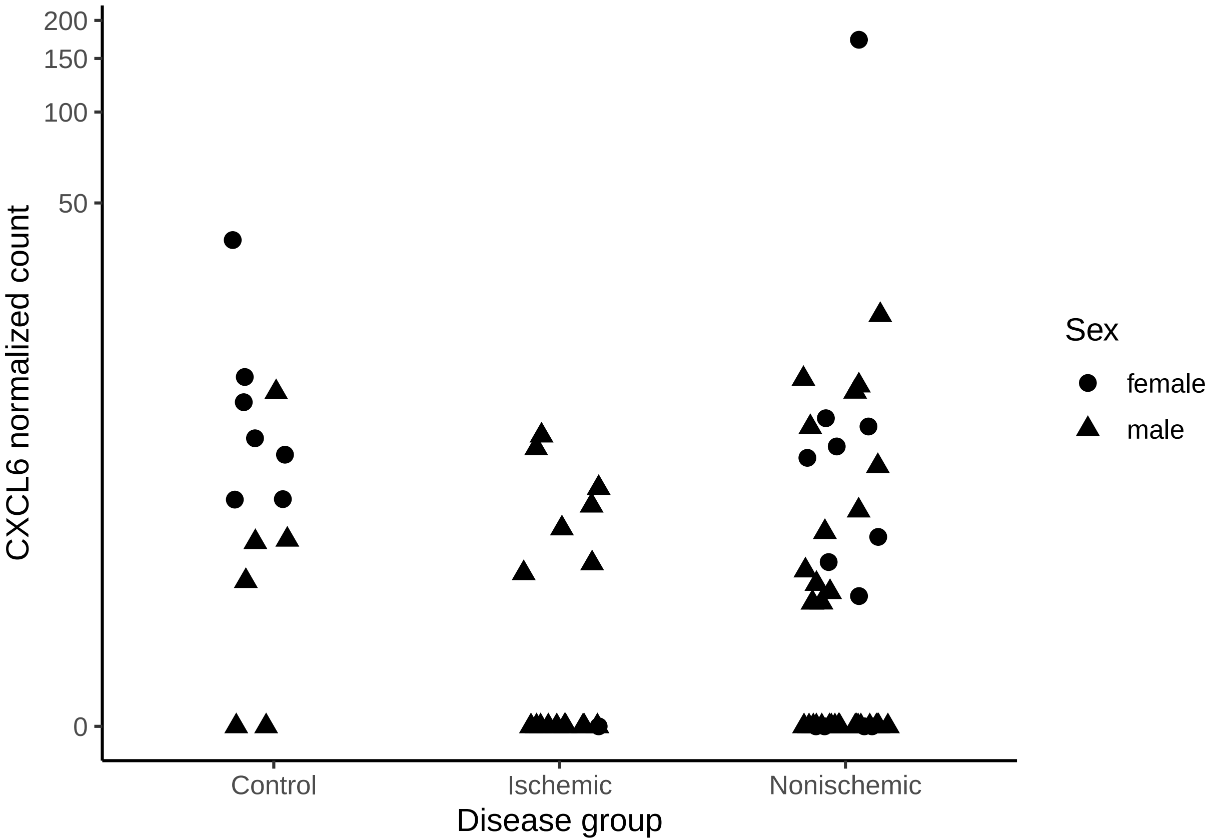

CXCL6 normalized count
200
150
100
50
0
Control
Ischemic
Nonischemic
Disease group
Sex
female
male
